# The Institutional Library

**Published:** 1921-06-18

**Authors:** 


### THE INSTITUTIONAL LIBRARY.

**Feeble-Mindedness in Children of School Age.** By C. Paget Lapage, M.D., M.R.C.P. Second edition. (Manchester: University Press. London: Longmans, Green & Co. 1920. Cr. 8vo. Pp. xv. and 309; Fourteen plates. 10s. 6d. net.)

This is a second edition of a very useful little book which appeared first in 1910. In the ten years which have elapsed since the appearance of the original edition much work has been done among mental defectives, and their position, as pathological entities requiring continual and skilled care, is daily becoming more clearly defined. The passing of the Mental Deficiency Act in 1913 was another forward step, and this little book explains the objects of this Act, and gives details of the various agencies interested in the work. The years of war have delayed the appearance of this edition, and, we feel sure, have also delayed some of the schemes devised to improve the lot of these patients.

There is not much change in the general arrangement of the book, and we were especially interested in the chapters on Diagnosis. As Dr. Lapage is a clinician of such wide experience we think his observations are of great value. Another useful feature is the chapter on the training and management of these children by Miss Mary Dendy, M.A., formerly Hon. Secretary of the Sandlebridge Colony at Alderley Edge, and now a member of the Board of Control.

We feel sure that, to those interested in the work, we can recommend this book as a compact and comprehensive little treatise.

**A Guide to Anatomy (for Students of Medical Gymnastics, Massage, and Medical Electricity).** By Miss E. D. Ewart. (London: H. K. Lewis & Co., Ltd. Price 16s. net.)

Embracing a fairly comprehensive survey of human anatomy systematically grouped, this textbook should find a place in the sphere of usefulness which the author had in view. The choice of textbooks needful in preparing for examinations in massage and medical gymnastics is by no means an easy matter. We believe that the work under review will be welcomed by teachers and students alike, for it is full enough to give the incipient masseuse the general knowledge of the subject she requires, while the special treatment of those sections which are particularly concerned in this branch of work adds to its usefulness. Apart from these features the book gives a good perspective view of the subject, and should provide excellent ground-work for the ambitious student who desires a more complete knowledge. As many practitioners of massage, medical gymnastics, and electricity also take up radiography the section on osteology could with advantage have been amplified by detailed reference to centres of ossification together with diagrams. The vascular and nervous systems are clearly elucidated. The old terminology has been adhered to throughout, though here and there alternatives are given.

**Gout.** By Llewellyn Jones Llewellyn, M.B. (London: Wm. Heinemann, Ltd. 1920. Pp. 469, xxviii. Price £1 10s. net.)

Dr. Llewellyn has added another considerable volume to the literature of gout. His book is the outcome of a close study of the disease as this is written in the contributions of his predecessors and as he has seen it pictured for himself in his own clinical experience. The history of the disease and of its medical relations has a classical attraction to which Dr. Llewellyn does full justice, and here it is interesting to note his opinion, not only that arthritic gout is becoming less prevalent, but that the type of the disease also has suffered attenuation. Nor does he allow that the *irregular* manifestations of the disease show any increase.

Dr. Llewellyn's discussion of the pathology and etiology of the disease and of the various hypotheses which have been advanced in explanation is most detailed and elaborate, and can be read with advantage by anyone interested in the subject. He himself places great stress on the influence of local foci of infection, and he argues his case with great skill and supports it by well-presented facts. The sections on treatment are particularly full and exact, and the subject of diet is dealt with in a really masterly fashion; such manifestations as lumbago, fibrositis, phlebitis, and other non-arthritic symptoms also receive due attention. A useful chapter on the ocular manifestations of gout is added by Mr. W. M. Beaumont. Altogether Dr. Llewellyn's book deserves to take rank with earlier treatises dealing with a disease which has through many years made a peculiar appeal to English physicians.

**The Art of High Health.** The Pilgrim's Books. (Philip Allan & Co. Price 5s. net.)

This is a reprint of four agreeable and semi-classical articles by Thomas Walker, a London stipendiary magistrate who died in 1836, and of Cornaro's treatise on long life written at the age of eighty-three in 1558. The series is intended for pocket companionship on walking or cycling tours, and is well got up in convenient size for this purpose. The ideas expressed by Walker are sometimes contradicted by the teachings of modern physiology, and the reader must guard against taking what he says for gospel. For rescuing both him and Cornaro from oblivion the "Graduate of Cambridge" who edits the series deserves our thanks.

**The Assessment of Physical Fitness.** By G. Dreyer, M.D., Professor of Pathology, Oxford University, in collaboration with G. F. Hanson, late American Medical Air Service. Large 8vo, pp. 116 + 11. (London: Cassell & Co., 1920. Price 10s. net.)

This little work, printed on good paper and in a French-looking type, aims at providing us with a human physical norm as regards weight, trunk length, chest circumference, and vital capacity. A brief but sufficient explanation precedes the tables, for males and females separately, since it is stated that physique differs according to sex in almost every particular. The measurements above mentioned are taken because these four have been found by Professor Dreyer to have definite inter-relationships. We miss here allusion to biometrical literature, as also (this neglect is about the only fault of a businesslike manual) to the recent work of Brugsch. Vital capacity is ascertained by an improved form of the spirometer of Hutchinson, to whom the book is dedicated. How many know that in Hutchinson's work a footnote states that the famous Sir Humphry Davy had a chest measurement of but twenty-nine inches? This suggests that C3 may bear very differently physically and intellectually!

